# An Objective Analysis of the Quality and Readability of Online Information on Breast Implant Illness

**DOI:** 10.7759/cureus.82042

**Published:** 2025-04-10

**Authors:** Taylor Blount, Cameron Gerhold, Virginia Bailey, Michael J Sweeney

**Affiliations:** 1 Plastic Surgery, Florida State University College of Medicine, Tallahassee, USA; 2 Orthopedic Surgery, Florida State University College of Medicine, Tallahassee, USA; 3 Clinical Sciences, Florida State University College of Medicine, Tallahassee, USA

**Keywords:** breast implant illness, explantation surgery, flesch-kincaid, google, health literacy, online patient education, readability measures

## Abstract

Breast implant illness (BII) has emerged as a significant concern for patients with breast implants, characterized by a range of symptoms, including fatigue, cognitive impairment, and joint pain. The growing recognition of BII has prompted increased scientific investigation. However, despite the rising prevalence of breast augmentation procedures, there remains a lack of consensus within the medical community regarding the diagnosis, etiology, and treatment of BII.

This study aimed to evaluate the readability of online resources pertaining to BII to determine their accessibility to the general population. A systematic internet search was conducted using three major search engines (Google, Bing, and Yahoo) with the query "breast implant illness." After excluding duplicates and paid content, 28 unique websites were analyzed using five established readability indices: Flesch-Kincaid Reading Ease Score (FRES), Gunning-Fog Index, Coleman-Liau Index, Simplified Measure of Gobbledygook (SMOG), and Automated Readability Index.

The results revealed a considerable range in the readability of online materials related to BII. The average FRES was 50.4, categorizing the material as "fairly difficult" to read. The Gunning-Fog Score averaged 9.3, suggesting a 9th-grade education level for comfortable comprehension. The SMOG Index indicated a reading level appropriate for individuals with at least 13 years of education. The Coleman-Liau Index suggested a reading level corresponding to a 21-year-old, while the Automated Readability Index pointed to a high school freshman level.

These findings highlight a significant disparity between the readability of available information and the recommended standards set by the American Medical Association and the National Institutes of Health, which propose that patient education materials should be written at a 6th-grade reading level or lower. The study's results are consistent with research on the readability of online health information in other domains, identifying a mismatch between the reading level of the general public and the complexity of available health information.

The importance of readability in online health information cannot be overstressed, particularly in the context of BII, where decisions may include elective surgery with inherent risks. The study calls for the development and implementation of standardized guidelines for creating online health materials that are accessible to a diverse audience. This includes simplifying language, using clear formatting, and incorporating visual aids to enhance comprehension.

## Introduction

Breast implant illness (BII) has gained significant attention in recent years due to the increasing recognition of its impact on patients with breast implants ​[1]. This syndrome is characterized by a constellation of symptoms such as fatigue, decreased focus, hair loss, joint pain, myalgias, arthralgias, and cognitive impairment, which often prompt patients to seek an explanation for their breast implants ​[2-4]. The association between the number of symptoms reported prior to explanation and the number of symptoms resolving following explanation highlights the significant impact of breast implant illness on patients' well-being [​4​]. Furthermore, the lack of well-defined diagnostic criteria and the highly heterogeneous presentation of breast implant illness emphasize the complexity of this condition ​[5,6].

Current events, such as the World Health Organization's recognition of breast implant-associated anaplastic large cell lymphoma (BIA-ALCL), have prompted scientific investigations and increased attention to understanding breast implant illness [7]. Additionally, the systemic symptoms of breast implant illness have been identified as a major risk factor for eventual surgery for explantation of the breast implant, further underscoring the impact of this syndrome on patients' health and well-being ​[2]. Despite increasing patient reports and social media attention, the medical community has yet to reach a consensus on the criteria for diagnosis, etiology, and treatment pathways for BII. This gap in knowledge has led many patients to seek information from the internet.

Online resources have become a pivotal source of health information for the public. They serve as the first point of contact for many individuals seeking to understand their health concerns [8]. However, the readability and quality of this information can vary drastically, which may influence patient decisions and outcomes.

Readability is a measure of how easy a text is to read. The readability of health information is particularly important because it directly impacts a patient's ability to understand their condition and the associated risks, benefits, and alternatives to proposed treatments ​[9,10]. In the case of BII, where misinformation can lead to undue anxiety or inappropriate medical decisions, the clarity of information is paramount.

As the prevalence of breast augmentation continues to rise, with over 300,000 procedures performed annually in the United States alone ​[11], the potential impact of BII becomes more significant. Thus, ensuring that patients can access and understand relevant health information is critical.

This study aims to evaluate the readability of online resources pertaining to BII to determine if they are accessible to the general population. This research highlights areas where improvements can be made to aid patient comprehension and facilitate informed decision-making.

## Materials and methods

To assess the availability and quality of information on BII, a systematic internet search was conducted. Recognizing that the internet is a primary source for patients seeking health information, we chose three major search engines (Google, Bing, and Yahoo) to reflect a broad spectrum of public access points.

On November 1, 2023, searches were performed using the query "breast implant illness" to capture the most relevant and frequently accessed online materials. To simulate the experience of an average user, all personalization features such as user data, cookies, and location information were disabled. Twenty websites were selected from each platform. The initial search yielded 60 websites, from which duplicates and sites requiring payment were excluded, resulting in 28 unique resources for analysis.

These resources included patient forums, educational websites from medical organizations, news articles, and informational pages from plastic surgery practices. The selected online materials were then subjected to a readability assessment using five established readability indices. Each index provides a different perspective on readability, considering factors such as word length, sentence complexity, and overall text structure.

Flesch-Kincaid Reading Ease Score (FRES)

This tool assesses text on a 100-point scale. It was developed by the US Military and has been extensively endorsed and utilized ​[12]. The higher the score, the easier it is to understand the document. This score can be evaluated as follows: 0-29 (very difficult postgraduate), 30-49 (difficult college), 50-59 (fairly difficult high school), 60-69 (standard eight to ninth grade), 70-79 (fairly easy seventh grade), 80-89 (easy fifth to sixth grade), and 90-100 (very easy fourth to fifth grade).

The formula to calculate the FRES is 206.835 - 1.015 × (average number of words per sentence) - 84.6 × (average number of syllables per word).

Gunning-Fog Index

This score utilizes the number of words, number of sentences, and number of complex words (≥3 syllables) to provide an estimate of the number of years one would need to spend in formal education to understand a text upon first reading ​[13].

The formula to calculate the Gunning-Fog Index is 0.4 * ((words/sentences) + 100 * (complex words/words)).

Coleman-Liau Index

This tool calculates the U.S. grade level of the text. For example, a score of 10 would equate to the text being written a 10th-grade reading level.

The formula to calculate the Coleman-Liau Index is 5.89 x (characters/words) - 0.3 x (sentences/words) - 15.8.

Simplified Measure of Gobbledygook (SMOG)

SMOG scores predict the years of education required to understand a piece of writing by taking into account the number of polysyllabic words (≥ 3 syllables per word)​ [14].

The formula to calculate the SMOG score is 1.043 * sqrt(number of words with 3+ syllables * (30/number of sentences)) + 3.1291.

Automated Readability Index

This tool takes into account the number of characters and words within a given text when determining how complex or readable a written material is ​[15].

The formula to calculate the automated readability index is 4.71 * (characters/words) + 0.5 * (words/sentences) - 21.43.

## Results

The assessment of the 28 websites using the five readability indices yielded a comprehensive overview of the accessibility of online information regarding BII. 

Flesch Kincaid Reading Ease Score 

FRES averaged 50.4 across the sampled content, categorizing the material as "fairly difficult" to read (Table 1). This score suggests that the average reader may encounter some difficulty understanding the information provided about BII without prior medical knowledge. Furthermore, the Flesch-Kincaid Grade Level indicated that the average readability level was akin to that of an 8th-grade student (Table 2).

**Table 1:** Readability Scores for Each Respective Measure

Numeric Scoring Index (0-100)	Mean ± SD	Rating
Flesch-Kincaid Reading Ease	50.4 ± 8.2	Fairly difficult

**Table 2:** Grade-Level Scoring Index GFS Score: Gunning-Fog Score, SMOG Index: Simplified Measure of Gobbledygook Index, CLI: Coleman-Liau Index, ARI: Automated Readability Index

Grade-Level Scoring Index	Mean ± SD	Age, y
Flesch-Kincaid Grade Level	8.3 ± 1.6	14
GFS Score	9.3 ± 2.1	15
SMOG Index	7.3 ± 1.3	13
CLI	15.2 ± 1.6	21
ARI	7.7 ± 2.2	14

Gunning-Fog Score

In contrast, the Gunning-Fog Score, which generally rates higher than the Flesch-Kincaid Grade Level due to its emphasis on word complexity, averaged 9.3, implying that the reader would need approximately a 9th-grade education to understand the text comfortably on the first reading.

Simplified Measure of Gobbledygook Index

The SMOG Index, designed to estimate the years of education needed to understand a piece of writing, suggested a reading level appropriate for individuals with at least 13 years of education, which would translate to someone in the first year of college in the United States.

The Coleman-Liau Index

The Coleman-Liau Index, which is less influenced by the use of frequently recognized words and more by characters per word, suggested a reading level that corresponds to a 21-year-old, which is indicative of college-level material. 

Automated Readability Index

The Coleman-Liau Index, along with the Automated Readability Index (ARI), which also suggested a reading level suitable for a high school freshman, points to a considerable disparity in the accessibility of information provided across different platforms.

The detailed results of the readability indices for each website were as follows: (i) The highest FRES was recorded at 70.3, which is considered relatively easy to read, while the lowest was at 30.2, indicating very difficult content; (ii) The Gunning-Fog Index ranged from 7.2 to 11.5, with higher values reflecting increased complexity; (iii) SMOG scores varied from 10 to 16, with a similar implication of reading difficulty as the Gunning-Fog Index; (iv)The Coleman-Liau Index presented a range between 10.5 and 14.2, suggesting that some materials were significantly more complex than others; (v) The ARI varied from 6.8 to 10.4, echoing the Flesch-Kincaid Grade Level in suggesting a middle to high school level of comprehension.

**Figure 1:**
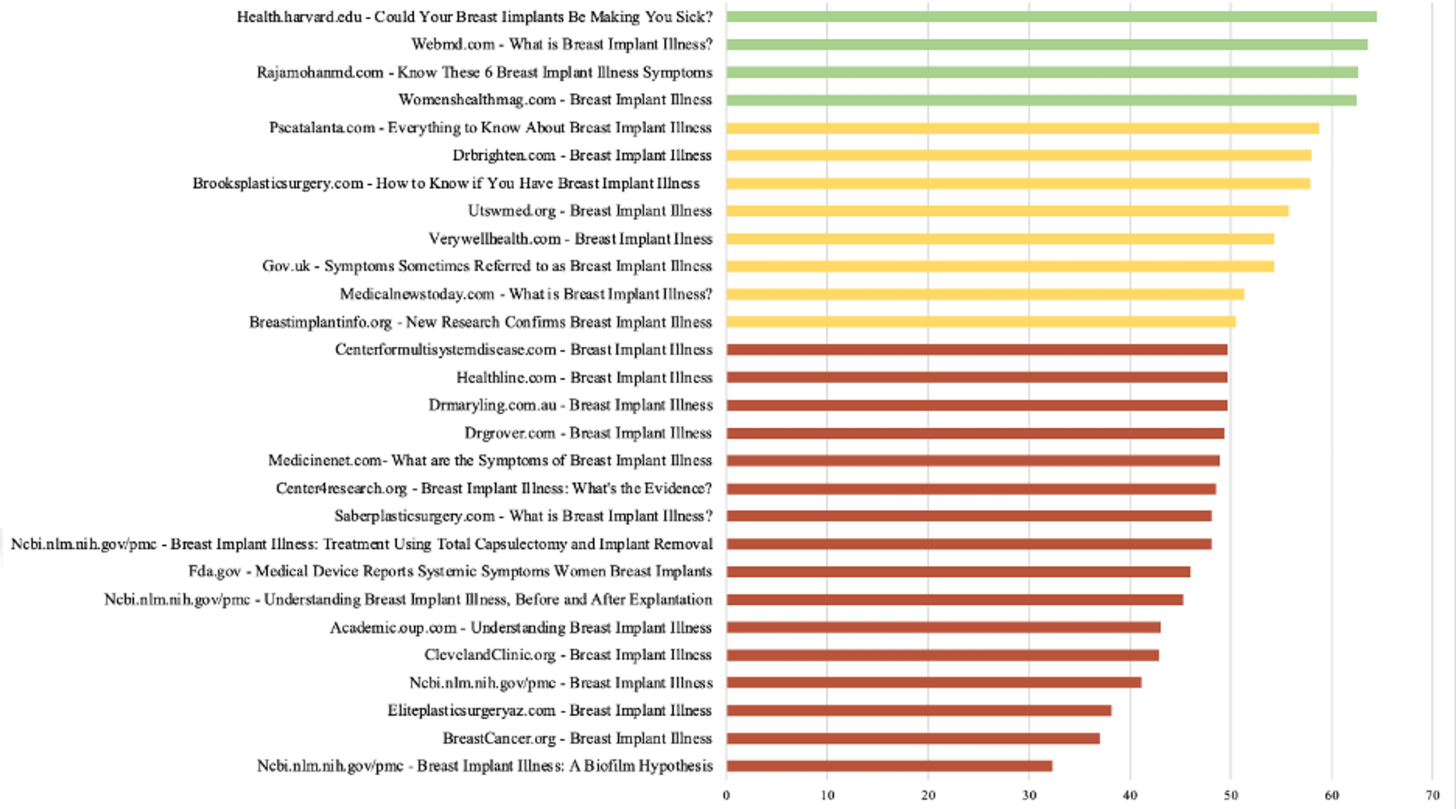
Flesch-Kincaid Reading Ease Scores by Website Based on a 0-100 score. A high score means the test is easier to read.

These findings suggest that while some online resources on BII are accessible to a wider audience, others may be prohibitive due to their complexity and the higher level of literacy required for comprehension (Figure 1).

## Discussion

The results of this study have illuminated a considerable range in the readability of online materials related to BII. This variability is a cause for concern as it suggests that much of the information available may not be accessible to all individuals seeking guidance on this condition. The disparity in readability levels is particularly troubling given the potential for misinformation to lead to patient anxiety and suboptimal medical decision-making.

The high Coleman-Liau Index scores observed in some resources indicate that many sites may be inadvertently excluding a significant portion of the population that does not have a college-level education. Given that approximately 19% of U.S. adults read below a 5th-grade level, it is imperative that medical information is presented in a manner that is understandable to the lay public [16].

The findings of this study suggest that online materials on BII are not meeting the recommended readability standards set by the American Medical Association and the National Institutes of Health, which propose that patient education materials should be written at a 6th-grade and 8th-grade reading level, respectively, or lower [17,18].

This study's results are consistent with research on the readability of online health information in other domains, which has similarly identified a mismatch between the reading level of the general public and the complexity of available health information [19,20]. Such a discrepancy highlights the need for the development and implementation of standardized guidelines for the creation of online health materials.

The importance of readability in online health information cannot be stressed enough. Clear and accessible information is essential to support patient autonomy and empower individuals to make informed decisions about their healthcare. In the context of BII, where the decisions may include elective surgery with inherent risks, the stakes are particularly high.

Health organizations and professionals producing online content should prioritize the creation of materials that are accessible to a diverse audience. This not only includes simplifying the language but also involves the use of clear formatting, bullet points, and visual aids to enhance comprehension. Visual aids, such as pictures, videos, and infographics, can restructure available information to potentially aid patients with understanding and retaining online materials since this avenue allows information to be presented in a more engaging format. Patient education materials should be designed with the reader in mind, ensuring that individuals of all literacy levels can benefit from the information provided.

This study's investigation into the readability of online resources on BII has highlighted a critical area of concern in patient education and health literacy. The findings demonstrate that the accessibility of information varies widely, potentially impacting patients' ability to make informed decisions about breast implants.

To address these disparities, it is essential to establish and adhere to standardized guidelines for the creation of online health information. These guidelines should prioritize simplicity, clarity, and the use of plain language to ensure that all individuals, regardless of their educational background or literacy level, have equitable access to important health information.

By improving the readability of health information, we can enhance patients' understanding of BII and its potential implications. This, in turn, promotes informed decision-making, which is a cornerstone of patient-centered care and autonomy. Moreover, accessible information serves to foster trust between patients and healthcare providers, which is essential for the effective management of any health condition.

Limitations

This study has several limitations that should be considered when interpreting the results. Firstly, the sample size and selection process may not fully represent the vast array of online information available on BII. The analysis was limited to 28 unique websites selected from the top results of three major search engines. This approach, while systematic, potentially excluded relevant resources that were not highly ranked by these search engines, thereby limiting the comprehensiveness of our analysis. Additionally, the study focused exclusively on English-language resources, which restricts the generalizability of our findings to non-English speaking populations. This language limitation is important to note given the global nature of both internet usage and the prevalence of breast implants. Lastly, although peer-reviewed journal articles may be among the first search results on a web browser, not all publications are accessible to the general public due to the subscription fees required for access to select journals. This reduces the amount of accurate, relevant information produced by experts and available to the public. Future research could address these limitations by expanding the sample size, incorporating a more diverse range of online resources, and including multilingual content to provide a more comprehensive assessment of BII information accessibility across different languages and cultures.

## Conclusions

The findings from this study call for a concerted effort to improve the readability of online health information about BII. Such efforts will not only benefit patients looking for information on BII but also set a precedent for the presentation of online health information on other conditions. As the internet continues to be a primary source of health information for the public, ensuring that this information is accessible and comprehensible becomes increasingly important. It is the responsibility of healthcare professionals, content creators, and health organizations to ensure that the information they provide can effectively reach and inform the intended audience, thus playing a critical role in enhancing health literacy and patient care outcomes.
